# A Systematic Model Specification Procedure for an Illness-Death Model without Recovery

**DOI:** 10.1371/journal.pone.0123489

**Published:** 2015-04-13

**Authors:** Christine Eulenburg, Sven Mahner, Linn Woelber, Karl Wegscheider

**Affiliations:** 1 Department of Medical Biometry and Epidemiology University Medical Center Hamburg-Eppendorf, Hamburg, Germany; 2 Department of Gynecology, University Medical Center Hamburg-Eppendorf, Hamburg, Germany; Queen's University Belfast, UNITED KINGDOM

## Abstract

Multi-state models are a flexible tool for analyzing complex time-to-event problems with multiple endpoints. Compared to the Cox regression model with a single endpoint or a summarizing composite endpoint, they can provide a more detailed insight into the disease process. Furthermore, prognosis can be improved by including information from intermediate events occurring during the course of the disease. Different model variants, options and additional assumptions provide many possibilities, but at the same time complicate the implementation of multi-state techniques. So far, no guiding literature is available to specify a multi-state model systematically. The objective of this work was to set up a general specification procedure for an illness-death model that optimizes the model fit and predictive accuracy by stepwise reduction of the model. As an application example, we reanalyzed data from an observational study of 434 ovarian cancer patients with progression as intermediate and death as absorbing state. The technique is described in general terms and can be applied to other illness-death models without recovery. The clock-reset approach was used, implicating that the time was reset to zero after progression. The non-homogeneous semi-Markov characteristic stated that the present time as well as the time between surgery and progression influenced survival after progression. Covariate effects on transitions were estimated and proportionality of transition baseline hazards was tested. The finally developed model optimized the accuracy of predictions for two simulated patients. This stepwise procedure yields parsimonious but targeted multi-state models with well interpretable coefficients and optimized predictive ability, even for smaller data sets.

## Introduction

Multi-state models are a flexible tool for analyzing complex disease processes, where individuals are allowed to move between a finite number of states. The states may be defined through the stages of the disease, incidences of clinical symptoms or occurring complications, or death. The states and the possible transitions between these states fully characterize the disease process. In recent years, multi-state models have been studied widely [1–9] and clinical applications have become more frequent [10,11]. Multi-state models are an extension of the classical survival model, usually solved by the Cox proportional hazard model [12,13]. There are two major advantages of multi-state models: Firstly, they can provide a detailed insight into disease processes, as covariate effects on each transition can be estimated. Etiological aspects of different phases of the disease can be studied. Analysis of competing states is possible, such as different causes of death or competing therapy outcomes (competing risk models) or a sequence of states such as disease recurrences (classical multi-state models). Secondly, prognosis from multi-state models can be more accurate than from the standard model with one single, potentially combined, endpoint. During the course of the disease, predictions can be adjusted when additional information like the occurrence of intermediate events becomes present.

In return for these advantages, the drawback of statistical instability has to be accepted. Dividing a process into multiple sub-processes bears the risk of small event counts within some of the studied transitions which in turn can result in unstable estimates with large confidence intervals and p-values. During the past years, multi-state models have been improved by allowing additional assumptions that can stabilize the results [2,7,8]. Various extensions and assumptions provide high flexibility, but at the same time complicate the specification of a multi-state model. Applications using these extensions are rare in the literature [7,11,14,15]. One cause may be uncertainty among investigators how to specify a multi-state model using these techniques. The purpose of our work was therefore to provide a formal instruction to specify an optimized multi-state model using the established options. The described adaptation procedure was modeled after a technique by P.F. Thall and J.M. Lachin [16]. Within another context, they described a formal model specification process for reducing stratified proportional hazards models. We expanded this Thall-Lachin-approach and transferred it into the multi-state context.

In the further work, we call the optimized models as restricted or reduced multi-state models. For a clinical example on ovarian cancer patients, we adapt a restricted multi-state model stepwise, following a defined procedure that starts with a full multi-state model with a minimum of restrictions. Modeling results and prognoses for two simulated patients will be outlined for every specification step. Our example shows that even for smaller data sets with rare event counts, multi-state models can improve estimation results and increase predictive accuracy.

## Methods

### Ethics statement

This study was approved by the Medical Board Hamburg, reference number #190504. All clinical investigations have been conducted according to the Declarations of Helsinki. Written informed consent was obtained from all patients to access their tissue and review their medical records when they first attended the clinic according to our investigational review board and ethics committee guidelines. Prior to analysis, patient information was anonymized and de-identified.

### Data example

Our data set covers the patients with epithelial ovarian cancer with primary surgery at the University Medical Center Hamburg-Eppendorf between 1993 and 2010. The anonymized and de-identified data used to produce the presented results can be downloaded from http://dx.doi.org/10.6084/m9.figshare.1248873.

Patients with neoadjuvant treatment (n = 50) before surgery were excluded to ensure comparability. Furthermore, patients with unknown progression status were excluded (n = 9). Patients with missing residual tumor status were recoded into no residual tumor (n = 8). Clinical details are described elsewhere [17]. 434 eligible patients with a median follow-up of 34.5 months (range 0.1–187 months) remained for analysis. To keep the example as simple as possible, only the three most important prognostic covariates are considered, i.e. the patients’ age at diagnosis, existence of a residual tumor after primary surgery and dichotomous staging of disease using the originally four-class FIGO (**F**édération **I**nternationale de **G**ynécologie et d'**O**bstétrique) classification scheme: low stage refers to FIGO i/ii; high stage represents FIGO iii/iv. Descriptive statistics and frequencies of observed transitions are summarized in Table 1.

**Table 1:** Frequencies of categories and of observed transitions.

	Total	Transition			
		Staying progression- free	Progression	Death without progress	Death after progression
		0→0	0→1	0→2	1→2
**Total** (% of all patients)	434 (100%)	129 (29.7%)	285 (65.7%)	20 (4.6%)	214 (49.3%)
**Residual tumor** (% within transition)	129 (29.7%)	10 (7.7%)	113 (39.7%)	6 (30.0%)	98 (45.8%)
**FIGO iii/iv** (% within transition)	342 (78.8%)	68 (52.7%)	260 (91.2%)	14(70.0%)	193 (90.2%)
**Age in years** median (range) within transition	59 (21–90)	55 (24–88)	60 (21–88)	62 (46–90)	62 (26–87)

### The multi-state model

**Fig 1:**
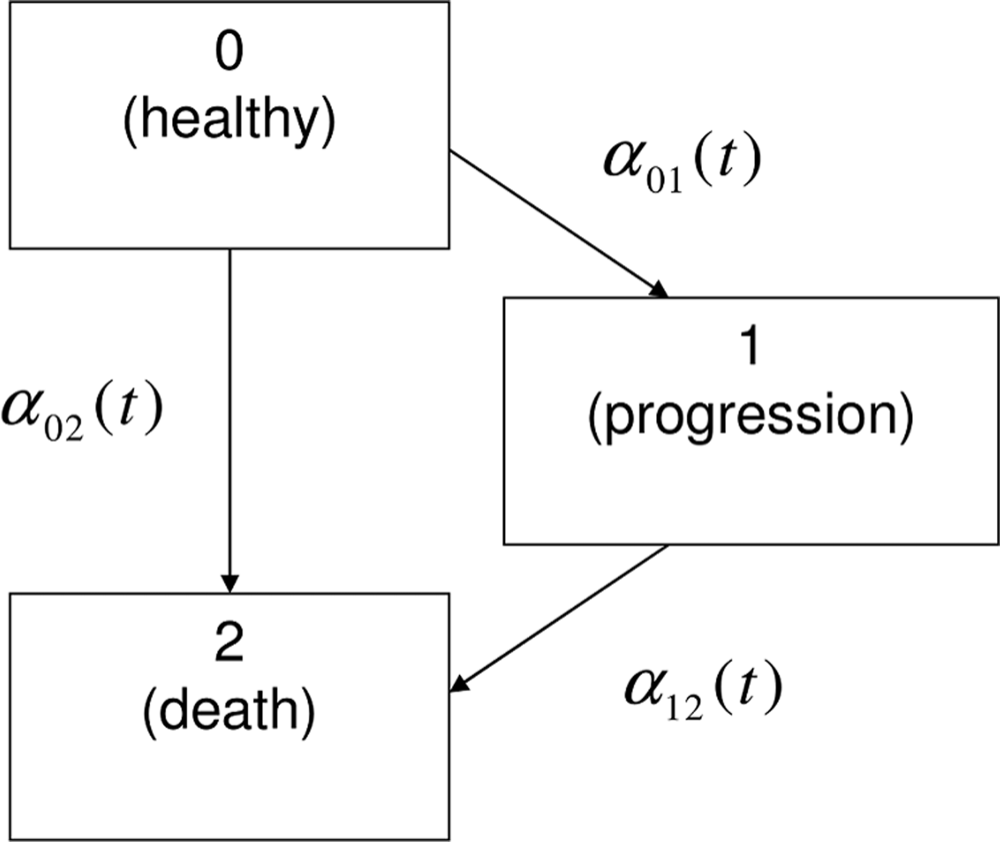
The illness-death model without recovery.

We define three states for our data example, differentiating the states after surgery: 0 or "healthy", 1 or "progression", and 2 or "death". All patients start in the "healthy" state, some of them move to "progression" and some patients transit directly to "death". It is also possible to move from "progression" to "death". In Fig 1, the multi-state model is displayed in a typical manner: boxes represent the states and arrows symbolize the possible transitions.

A model with the structure given in Fig 1 is generally called an illness-death model or a disability model without recovery. It is a frequently approached example of a multi-state model [3,18–20]. The transitions are quantified by hazard rates α_ij_ (t) of patients to leave state i for state j. The hazard can be understood as instantaneous potential of a specific transition at time t. For each transition, a Cox proportional hazards model [12,13] can be used to link the transition-specific hazard functions α_01_ (t),α_02_ (t) and α_12_ (t) with potentially prognostic factors. In multi-state theory, there are two ways to define the time t in α_ij_ (t). With the "clock-forward" approach, the time refers to the time since study start. The clock continues running, independent of the occurrence of intermediate events. In contrast, the "clock-reset" approach assumes a reset to zero, every time the subject moves to another state. The current time t then refers to the sojourn time in the present state [8]. For that case, we redefine new time scales t^0^ and t^1^, depending on the leaving states 0 = "healthy" and 1 = "progression".

A property that is often assessed in multi-state modeling is the Markov assumption. It implies that the future depends on the history of a process only through the present [4,21,22]. In clock-reset models the time scale itself depends on the time when the present state was reached, therefore the Markov assumption is violated by definition. Thus, only clock-forward models can meet the Markov assumption. The semi-Markov assumption [4,7,8] relaxes the Markov restriction: The process may depend on the present state and the time since entry of that state. Further dependence of the time since initiation makes the homogeneous semi-Markov model a non-homogeneous semi-Markov model. For the illness-death model, this implies that $\alpha_{12}({\text{t}}^{1},{\text{t}}_{01}^{0})$, where ${\text{t}}_{01}^{0}$ represents the sojourn time in the healthy state. Practically, ${\text{t}}_{01}^{0}$ is included as a covariate for the transition 1→2. Semi-Markov clock-reset models are often used when duration dependencies should be modeled.as they bear the advantage that information from the process history can be included as transition-specific explanatory covariates.

The non-homogeneous semi-Markov clock-reset model for the ovarian cancer example is specified through the transition hazards in (1),

$$\begin{array}{l} \alpha_{01}(t^{0})=\alpha_{01,0}(t^{0})\exp(\beta_{01,1}\;\cdot age+\beta_{01,2}\cdot res\;tum+\beta_{01,3}\cdot stage)\\ \alpha_{02}(t^{0})=\alpha_{02,0}(t^{0})\exp(\beta_{02,1}\;\cdot age+\beta_{02,2}\cdot res\;tum+\beta_{02,3}\cdot stage)\\ \alpha_{12}(t^{1},t_{01}^{0})=\alpha_{12,0}(t^{1})\exp(\beta_{12,1}\;\cdot age+\beta_{12,2}\cdot res\;tum+\beta_{12,3}\cdot stage+\beta_{12,4}\cdot t_{01}^{0}). \end{array}$$ (1)

The hazard for each transition i→j, I ≤ j ∈ {0,1,2}consists of a nonparametric baseline hazard function α_ij,0_(t) and a factor containing covariate effects, which may be also time-dependent. β_ij,c_ represents the transition-specific regression coefficient for covariate c. In (1), ten coefficients have to be estimated, three for each of the three transitions, plus one for ${\text{t}}_{01}^{0}$.

Sometimes the assumption of transition-specific covariate effects results in overfit, which may be a problem particularly in processes with rare event counts. In our data example, only 20 events are observed for the transition 0→2. The result is that estimates lose precision. Yet, multi-state models offer options for adapting more parsimonious models. With further assumptions on potentially proportional baseline hazards or identical covariate effects across transitions, covariate effects can be estimated more efficiently.

### The model specification process

The procedure starts with the full Markov model, where transitions and covariate effects are allowed to vary between transitions. Further steps result from answering the following questions:

1. What is the appropriate time scale for the baseline hazards?
2. Is the Markov assumption met?

Can we assume equal (*H*_*α*_ : *α*_*ij*,0_(*t*) = *α*_*kl*_,_0_(*t*), I,j,k,l ∈ {1,2,3}) or proportional (*H*_*γ*_ : *α*_*ij*,0_(*t*) = *α*_*kl*_,_0_(*t*). *e* ^*γ*^, I,j,k,l ∈ {1,2,3}) baseline hazard functions across transitions?

Can we assume some parameter effects to be identical (*H*_*β*_ : *β*_*ij*, *c*_ = *β*_*kl*_,_*c*_, I,j,k,l ∈ {1,2,3}) across transitions?

Finally, a tailor-made restricted multi-state model is obtained in a step-down procedure.

Choosing the appropriate baseline time scale may depend on the context as well as on practical considerations. Generally, in clinical or epidemiological studies, there are various possible time scales like the time since onset of a disease, time since surgery, time since a special treatment or time since birth. All these chronological time scales differ only in their origins. Major criteria for the choice of the time scale is the fact that its scale must be relevant in the investigated context to ensure the interpretability of the covariate effects [23]. The usability of different time scales depends on the application. In the present context, the time since primary surgery is considered.

In multi-state modelling, there are even more aspects to consider with respect to the time scale. While the clock-forward approach uses the time from onset of the study for all transitions, the observations start at time zero after each transition when the clock-reset approach is used. For illness-death models, this choice concerns only the transition from illness to death, since for transitions from the initial state the time since onset is the only available time scale. In this work, we proceed with the clock-reset approach, as it bears the advantage that the sojourn time spent in the healthy state before experiencing a progress may be explicitly included as a covariate for the transition from progression to death. For the ovarian cancer example, the non-homogeneous clock-reset semi-Markov multi-state model is specified according to Cox [12,13] through the transition hazards as in formula (1), with transition-specific baseline hazards α_ij,0_ and covariate effects β_ij,c_, i<j∈ {1,2,3} for covariate c. The Markov assumption is violated by construction as a clock-reset model is adapted. Otherwise, it could have been checked by testing the effect of the time in the healthy state ${\text{t}}_{01}^{0}$ on the transition from progression to death. Significance of ${\text{t}}_{01}^{0}$ would reveal that the Markov assumption should be rejected in favor of the semi-Markov assumption. Further, a non-parametric approach basing on Kendall's tau was developed for testing markovianity [24].

To make assumptions on the proportionality of baseline hazards, clinical considerations, literature statements, graphical and statistical tests should be considered. From a clinical point of view and as also literature states, it is reasonable to imply proportional baseline curves for transitions ending up in the death state, while transitions landing in progression follow a non-proportional baseline hazard [25]. The assumption can be tested using standard approaches, like the Schoenfeld test of proportional hazards or graphically plotting ln(cumulative baseline hazard) against ln(analysis time) for each transition. If the curves appear parallel, the assumption of proportional hazards holds.

To test whether covariate effects are identical across transitions, interactions between covariates and transitions are tested and eliminated stepwise. The likelihood ratio test and the AIC are used as specification criteria. If an interaction is insignificant, a version of the model omitting this interaction is recalculated. If the AIC of the latter model is improved and the likelihood-ratio test does not attest a significant difference between the models, the latter model is preferred. This step is repeated until the model contains only significant interactions. In a last step, insignificant factors will be waived stepwise.

For further comparison and evaluation, we examine prognostic features of the different models. We consider two simulated patients with opposite characteristics as in Table 2. Patient A is supposed to have a good prognosis, while patient B has a bad prognosis at onset.

**Table 2:** Characteristics of two simulated patients with good (patient A) and bad prognosis (patient B).

	Patient A	Patient B
Age in Years	<60	>60
Residual Tumor	no	yes
FIGO stage	i/ii	iii/iv
Months in the healthy state	24–48	≤12

Prognosis in semi-Markov models is not self-evident, as the time in the healthy state is not known at onset, but can be updated when the event occurs. It is included as prognostic variable for transition 1→2, but omitted for transitions 0→2 and 0→1. For the two simulated patients prognoses are computed at the essential steps through the adaptation process.

To evaluate which model performs best, the predictive ability will be compared using prediction error curves, a time dependent estimate of the Brier score. The time-dependent Brier score at time *t* is defined as the squared difference between the real survival status at time *t* (1 if subject is alive at *t*, 0 otherwise) and the prediction from time 0 of surviving *t*, which is model based. As the survival status at time *t* may be right censored for single observations, inverse probability of censoring weights (IPCW) are used [26,27]. The 0.632+ estimator of prediction error for survival data [28] is used, which is a linear combination of the downward biased apparent error and the upward biased bootstrap cross-validation estimator. The bootstrap cross-validation component is based on 300 samples of training sets with each 300 subjects and accordingly 134 subjects in the validation sets. Technical details of the method are described in [26–30].

For technical reasons, we subtracted half a month from the progression time in cases where progression and death coincided.

Models and graphs are calculated with Stata and the R package *mstate* and *pec [30–32]*. Theoretical aspects of the prognosis can be found in the accompanying literature [25,32] and in the works by Putter et al.[7,8].

## Results

Throughout the adaptation process, we use the clock-reset approach and start with the non-homogeneous semi-Markov model introduced in (1). Because of the chosen time scale and the fact that the time to progression ${\text{t}}_{01}^{0}$ impacts the transition 1→2 significantly, the Markov assumption is violated and replaced by the semi-Markov assumption (p<0.001). Further results of the non-homogeneous semi-Markov model with freely varying coefficients and with transition-specific baseline hazards (in the latter: the "full model") are summarized in Table 3.

**Table 3:** Results from the full semi-Markov multi-state model for distinct transitions.

****Covariate****	****HR**** ^a^	****Lower 95%-CI****	****Upper 95%-CI****	****p-value****
**Transition 0→1**				
Age	1.003	0.992	1.014	0.579
Residual tumor	1.991	1.551	2.555	<0.001
FIGO iii/iv	4.146	2.691	6.389	<0.001
**Transition 0→2**				
Age	1.044	1.006	1.084	0.021
Residual tumor	1.255	0.431	3.655	0.677
FIGO iii/iv	0.786	0.260	2.381	0.671
**Transition 1→2**				
Age	1.015	1.003	1.028	0.014
Residual tumor	1.713	1.282	2.287	<0.001
FIGO iii/iv	0.709	0.440	1.143	0.158
Time to progression	0.971	0.958	0.983	<0.001

^a^Hazard ratio

**Fig 2:**
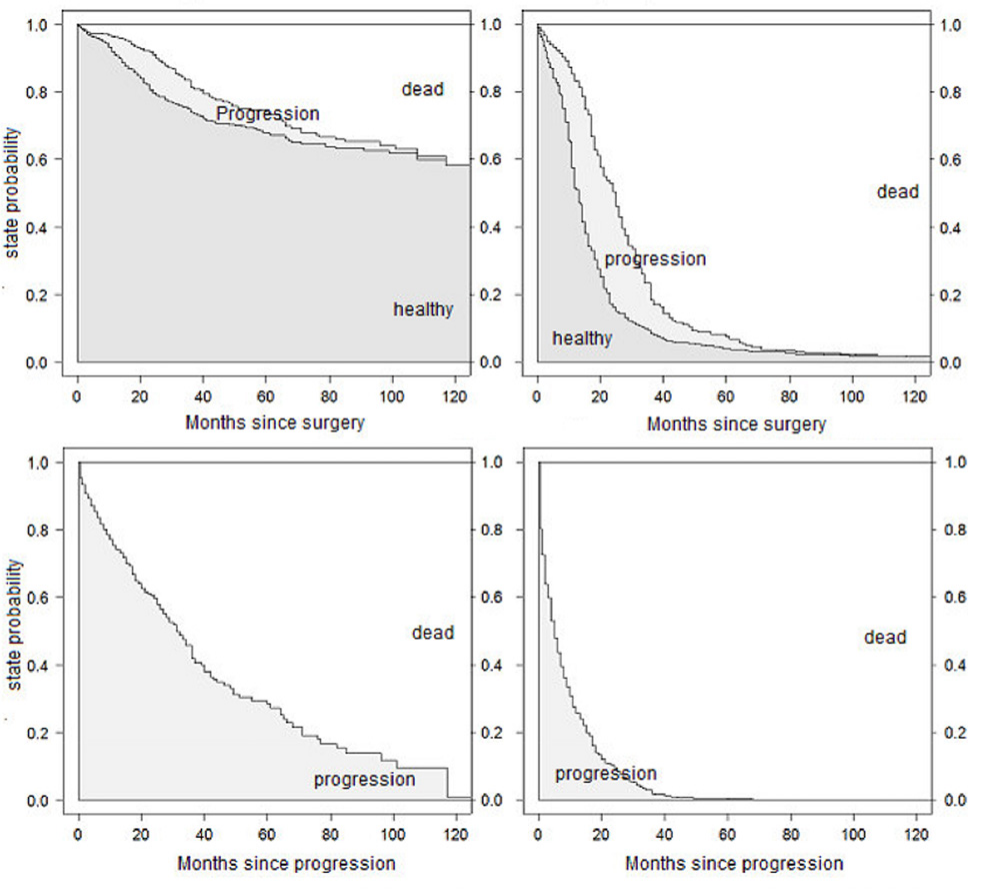
Predicted state probabilities from the full model. Probabilities for patients A (left) and B (right) for being in distinct states after study onset (upper) and immediately after progression (lower).

The state probabilities from the full model for patients A and B are shown in Fig 2. They can be interpreted as the probability of being in a particular state at a certain time after starting in a given state. Probabilities in Fig 2 are stacked, this means that the probability of being in each state is represented by the height of the corresponding band. The states are ordered by increasing severity, so that the probability of being alive can be simply read off by adding the neighbored gray belts. The two upper graphs show the state probabilities over time since study onset, starting in the healthy state. Of course, the probability of staying in the healthy state decreases from 1 at time 0. At the same time, the probabilities of progression and dying increase. It is visible that the probability of being in the state after progression decreases again around the 30th month, because these individuals further transit to the death state. Correspondingly, the lower graphs display the probabilities of staying in the progression state or dying after progression.

**Fig 3:**
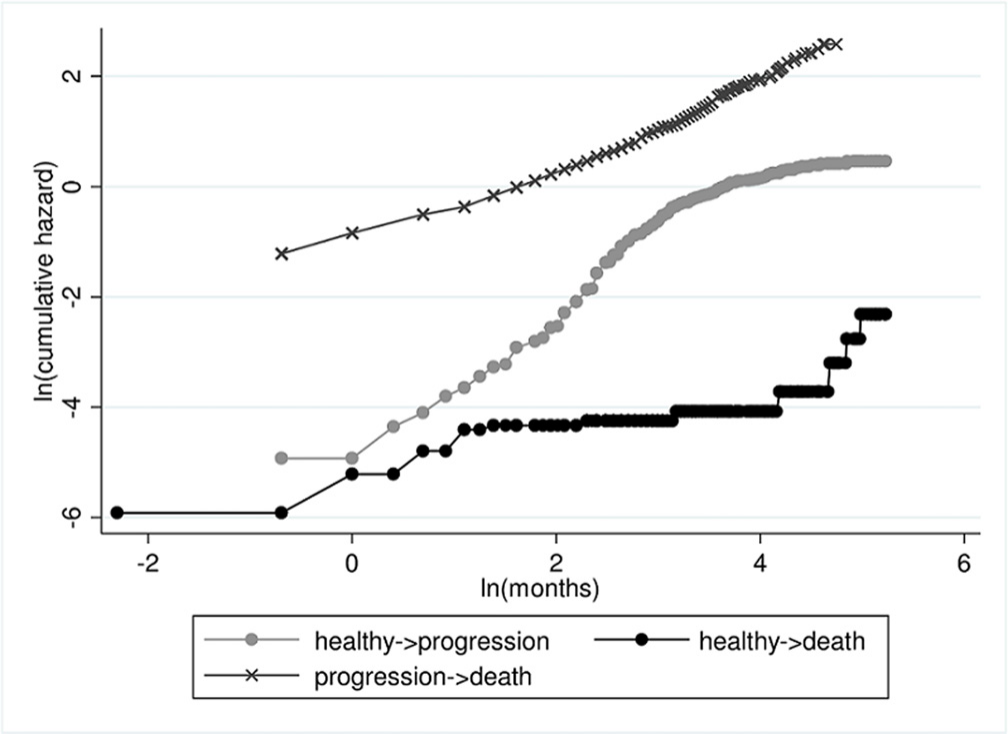
The log-log plot to test the assumption of proportional baseline hazards. Parallelism of the curves in a log-log plot indicates proportionality of the baseline hazard curves. This plot does not contradict the assumption of proportional baseline hazards for transitions into "death".

Next, we test the assumption of proportional baseline hazards of the transitions into "death". Schoenfeld's test does not attest a significant violation of the PH assumption concerning all three baseline hazard functions (transition 0→1 and transition 0→2: p = 0.086; transition 0→1 and transition 1→2: p = 0.731; transition 0→2 and transition 1→2: p = 0.068). But of course, this test depends on the sample size and may be underpowered, especially as the transition 0→2 has only 20 events. From the log-log plot in Fig 3 it is unclear whether the assumption holds for the death hazards. For proportionality, the curves have to be parallel. However, there seems to be no clear violation of the PH assumption either.

To exemplify the options of the multi-state model we assume proportional baseline hazards for mortality and an arbitrary baseline hazard for progression in this example.

Accordingly, hazards are specified through

$$\begin{array}{l} \alpha_{01}(t^{0})=a_{01,0}(t^{0})\exp(\beta_{01,1}\cdot age+\beta_{01,2}\cdot res\;tum+\beta_{01,3}\cdot stage)\\ \alpha_{02}(t^{0})=a_{02,0}(t^{0})\exp(\beta_{02,1}\cdot age+\beta_{02,2}\cdot res\;tum+\beta_{02,3}\cdot stage)\\ \alpha_{12}(t^{1},t_{01}^{0})=\exp(\gamma )\alpha_{02,0}(t^{1})\exp(\beta_{12,1}\cdot age+\beta_{12,2}\cdot res\;tum+\beta_{12,3}\cdot stage+\beta_{12,4}\cdot t_{01}^{0}). \end{array}$$ (2)

**Fig 4:**
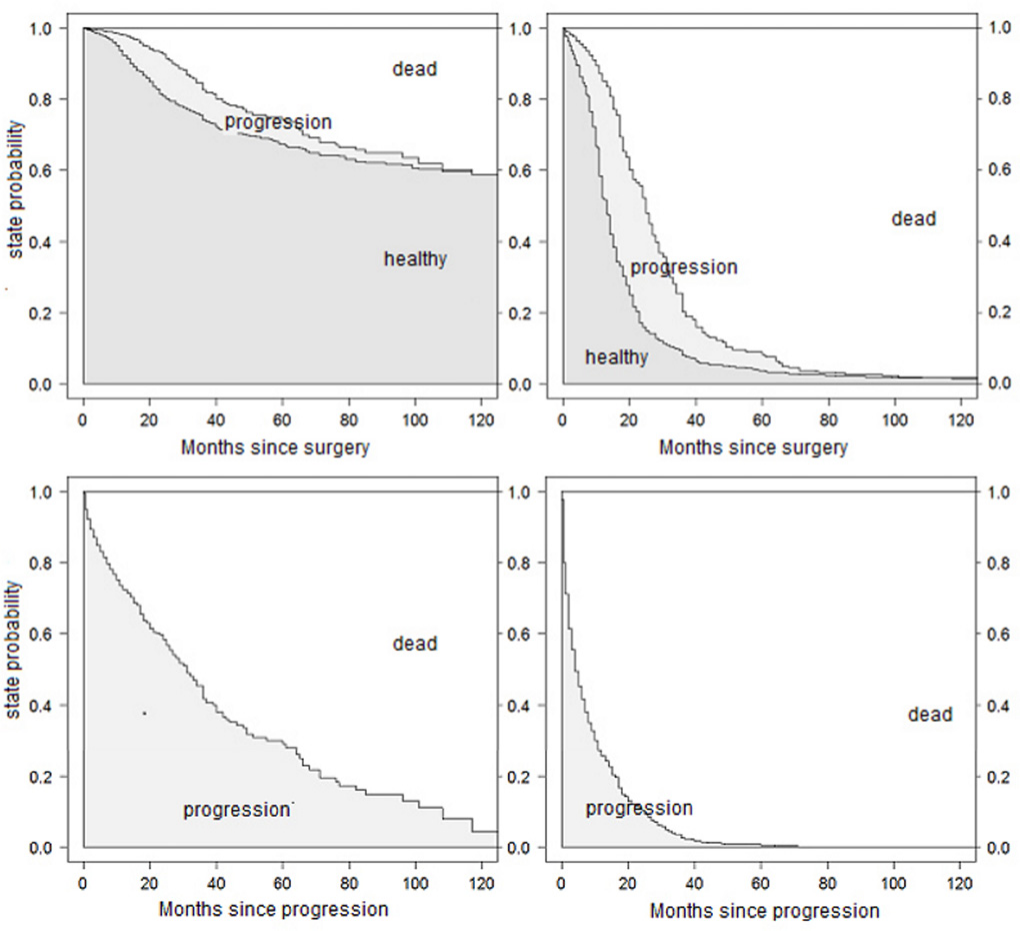
Predicted state probabilities from the PH model. Probabilities for patients A (left) and B (right) for being in distinct states after study onset (upper) and immediately after progression (lower).

In (2), the factor for proportionality between the baseline hazards for the transitions to death α_02_and α_12_ is denoted by exp(γ). It demonstrates how the risk of dying changes with occurrence of a progression. This semi-Markov model with proportional baseline hazards for transitions into "death" is called the "PH model" in the further text. Estimated hazard ratios from the PH model are reported in Table 4. The predicted state probabilities from the PH model for Patients A and B are displayed in Fig 4.

**Table 4:** Results from the PH model.

Covariate	HR^a^	Lower 95%-CI	Upper 95%-CI	p-value
**Transition 0→1**				
Age	1.003	0.992	1.014	0.579
Residual tumor	1.991	1.551	2.555	<0.001
FIGO iii/iv	4.146	2.691	6.389	<0.001
**Transition 0→2**				
Age	1.054	1.014	1.096	0.008
Residual tumor	1.578	0.544	4.579	0.401
FIGO iii/iv	1.042	0.355	3.061	0.941
**Transition 1→2**				
Age	1.015	1.003	1.027	0.015
Residual tumor	1.680	1.259	2.243	<0.001
FIGO iii/iv	0.712	0.442	1.145	0.161
Time to progression	0.972	0.960	0.984	<0.001
Progression	571.406	37.332	8745.913	<0.001

^a^Hazard ratio

To test whether covariate effects can be assumed to be identical across transitions, interactions between covariates and transitions are tested and eliminated stepwise. As in the previous model, we assume proportional baseline hazards of the transitions into "death". The PH model is the most general model considered. In the first step, equalizing the effect of residual tumour across all three transitions (model B) yields a reasonable model reduction regarding AIC and the likelihood ratio test. Assuming furthermore equal FIGO effects across all transitions does not improve the model. However, equal effects of FIGO staging across transitions into "death" (model C) increases the model fit. The effect of age should be modelled independently for the distinct transitions, according to our selection criteria. Regarding insignificant main effects in the next step, age is waived as predictor for the transition from "healthy" to "progression" (model D) due to insignificance. Finally, we waive the effect of FIGO staging for the transitions into "death" (model E). Table 5 shows the model fit criteria of the corresponding models.

**Table 5:** Adapting a parsimonious model: tests for the equality of covariates across transitions and for omitting covariates.

Model	Log-likelihood	AIC	df	LR-test^a^	p
**PH model**	-2640	5301	11		
**B**	-2640	5298	9	B vs. A	0.657
**C**	-2640	5297	8	C vs. B	0.506
**D**	-2641	5295	7	D vs. C	0.517
**E**	-2641	5295	6	E vs. D	0.158
				E vs. A	0.594

^a^Likelihood-ratio test

Following AIC, the most restrictive model E and model D provide a similar goodness of fit. The likelihood ratio test does not show significant differences between the reduced sub-models. We prefer the parsimonious model and finally specify the restricted multi-state model E. In this model, age is supposed to affect only the transitions into death and is not informative for progression. FIGO staging only correlates with progression and has no effect on transitions into "death". Residual tumour is supposed to have equal effects across transitions. The timing of progression impacts the prognosis after progression. The hazard functions for the final restricted multi-state model E are specified as in formula (3),

$$\begin{array}{l} \alpha_{01}(t^{0})=\alpha_{01,0}(t^{0})\exp(\beta_{1}\cdot res\;tum+\beta_{01,2}\cdot stage)\\ \alpha_{02}(t^{0})=\alpha_{02,0}(t^{0})\exp(\beta_{1}\cdot res\;tum+\beta_{02,3}\cdot age)\\ \alpha_{12}(t^{1},t_{01}^{0})=\exp(\gamma )\alpha_{02,0}(t^{1})\exp(\beta_{1}\cdot res\;tum+\beta_{12,3}\cdot age+\beta_{12,4}\cdot t_{01}^{0}). \end{array}$$ (3)

**Fig 5:**
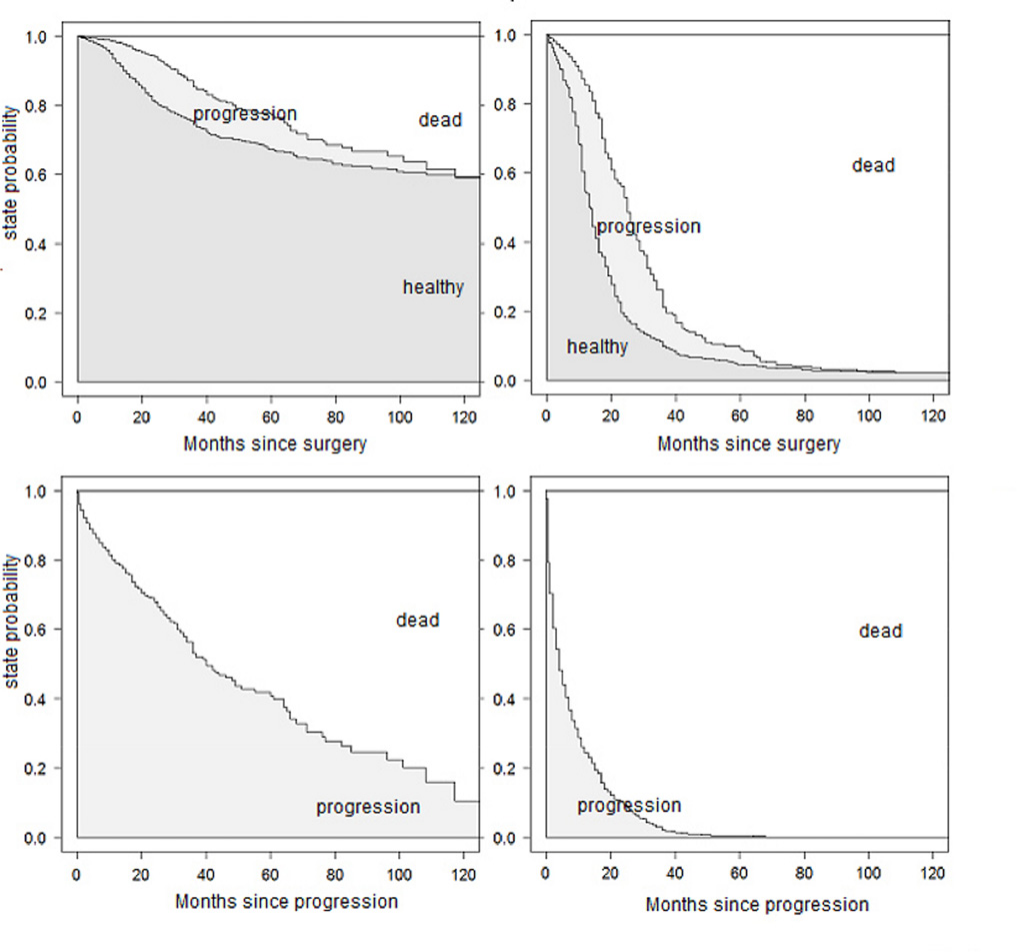
Predicted state probabilities from the reduced PH model. Probabilities for patients A (left) and B (right) for being in distinct states after study onset (upper) and immediately after progression (lower).

While ten coefficients have to be estimated in (1), the model in (3) gets along with only six. The estimated hazard ratios from model (3), in the following called the "reduced PH model", are presented in Table 6. The predicted transition probabilities for patients A and B are displayed in Fig 5.

**Table 6:** Results of the reduced PH model.

Covariate	HR^a^	Lower 95%-CI	Upper 95%-CI	p-value
**Transition 0→1**				
Residual tumor	1.807	1.507	2.167	<0.001
FIGO iii/iv	4.404	2.889	6.713	<0.001
**Transition 0→2**				
Age	1.054	1.014	1.096	0.008
Residual tumor	1.807	1.507	2.167	<0.001
**Transition 1→2**				
Age	1.014	1.002	1.026	0.023
Residual tumor	1.807	1.507	2.167	<0.001
Time to progression	0.973	0.961	0.985	<0.001
Progression	421.312	30.707	5780.573	<0.001

^a^Hazard ratio

**Fig 6:**
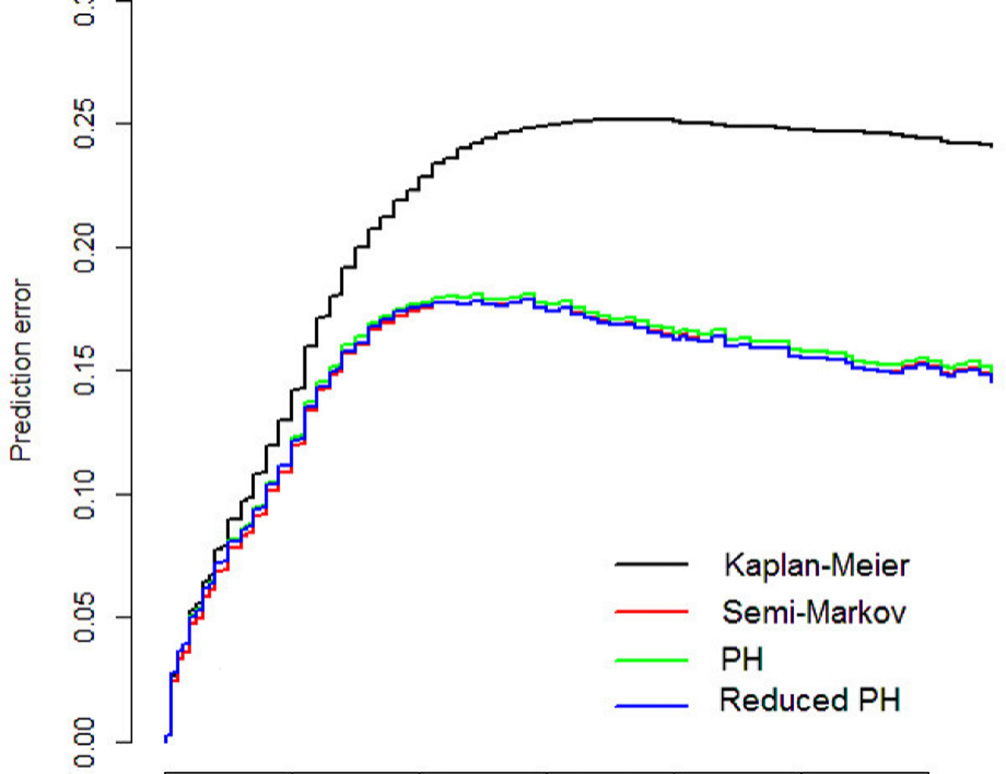
Prediction error curves. Time-dependent prediction errors for the null model (black), the full semi-Markov model (red), the proportional hazards model (green) and the reduced PH model (blue).

The prediction error curves of the three models and from a null model without covariates, the Kaplan-Meier model, are displayed in Fig 6. It is visible that the three adapted multi-state models have a better predictive ability than the null model. However, the full model, the PH model and the reduced PH model show a very similar performance. From the final model we summarize that the time to progression has a prognostic impact on survival after progression. Every progression-free month decreases the mortality by about 2.8% (95%-CI [0.015–0.039], p<0.001). Patients with high FIGO staging have an increased progression risk compared to patients with low FIGO staging (HR = 4.404, 95%-CI [2.889–6.713], p<0.001). A residual tumor increases the risk for progression and death (HR = 1.807, 95%-CI [1.507–2.167], p<0.001). Age increases mortality before progression by 5.4% per year (95%-CI [0.014–0.096], p = 0.008) and after progression by 1.4% per year (95%-CI [0.001–0.026], p = 0.023). Mortality is highly increased after the occurrence of progression. Yet, we renounce to interpret the unstable effect estimate. It is encouraging that almost all confidence intervals are shortened compared to the full model.

## Discussion

We defined a systematic modeling procedure for reducing a semi-Markov multi-state model for illness-death situations without recovery. This is useful in particular for small data sets with low event counts for single transitions, as overfitting can be avoided and accuracy of predictions can be improved. As an application example, we adapted a reduced multi-state model to data from 434 ovarian cancer patients. The model specification process starts with defining the time scale and testing the markovianity of the process. A semi-Markov multi-state model is adapted using the clock-reset approach. Covariate effects vary freely across distinct transitions. The sojourn time in the healthy state is included as prognostic covariate for the survival time after progression.

Step by step, additional assumptions are made to reduce the model. Proportional baseline hazards for the transitions from "healthy" to "death" and from "progression" to "death" did not change the results crucially: Effects for the transition from "healthy" to "progression" remain unchanged, as this was not affected by the recent assumption. Estimates for the transitions into "death" are also similar to results from the full model, supplemented by a hazard ratio describing the change of the mortality after entering the "progression" state. This estimate is very unstable, as the large confidence interval suggests. The curves for the estimated state probabilities from the full model in Fig 2 and the PH model in Fig 4 look very similar.

Assumptions of equal covariate effects are tested with likelihood-ratio tests on interactions between transitions and covariates. The resulting reduced model assumes equal effects for residual tumor across transitions, varying age effects for death before and after progression, while progression is unaffected by age. FIGO staging is assumed to not affect transitions into death, but to increase the probability of progression. The time in the healthy state is supposed to impact survival after progression.

This final model improves the precision of the estimated hazard ratios by shrinking the confidence intervals of the parameter estimates. But there is a trade-off relation between gaining precision and inducing bias by further specification of the model through additional assumptions. This is confirmed in the present example, where the prediction error curves in Fig 6 are very similar for the full model, the PH model and the reduced model. As the prediction error curves refer to predictions from time 0 and since occurrence and timing of the intermediate event is not known at study onset, information on the intermediate event is only considered for transition 1→2 when a clock-reset approach is used. This curtails the predictive benefits of the multi-state models. However, the estimated state probabilities for selected scenarios and simulated patients in Fig 5 remain also more or less unchanged. This matter indicates that our assumptions are reasonable and the bias due to misspecification is small (if we assume that the semi-Markov model in (1) tells the truth!). All three model are equally right or wrong.

The restricted multi-state model optimally balances fit and parsimony. Especially in smaller data sets, reducing the model may be useful to receive meaningful and stabilized results. However, the flexibility of the model can cause problems. For the restricted multi-state model it is an essential requirement that model assumptions as well as considerations about the baseline time scales are selected very carefully, taking clinical facts into account. In our data, the time between onset and the date of progression as well as the sojourn time in the progression state affect the prognosis after progression. Therefore, the use of a clock-reset semi-Markov model was reasonable.

Inference from Markov- or semi-Markov models may be easier than from non-Markov models. However, for non-Markov models, Gunnes et al. [33] provide statistical solutions. Furthermore, the assumption of equal effects of a residual tumour on progression and death at any time are realistic from a clinical point of view. Progression is sometimes understood as a surrogate endpoint for overall survival in ovarian cancer. Accordingly, associations between predictive markers and the endpoints progression and death may be assumed to be similar [34].

Generally, multi-state models provide a flexible framework for understanding clinical events under consideration of the disease process as a whole, not only focusing on one single endpoint, like the classical Cox regression model does. Multi-state models are frequently used for investigations on cancer [7,11,35], leukaemia and bone marrow transplantation [5,15,36–39], joint replacements [10], HIV [40] and other fields [2,22,41]. Yet, the use of a reduced model with more modeling assumptions is scarcer. The presented detailed exemplification on how to specify a parsimonious model may help to further spread the use of restricted multi-state models.

Most multi-state models can be modeled with every software that can handle the Cox regression model. Specific programs are furthermore available, like the R package *msm* [42,43], or the *mstate* package [25,31,32] for semiparametric models including prognosis.
